# Supplementary material for: Phosphorylation of MAVS/VISA by Nemo-like kinase (NLK) for degradation regulates the antiviral innate immune response
Source: Nat Commun. 2019 Jul 19;10:3233. doi: 10.1038/s41467-019-11258-x (PMC6642205; doi:10.1038/s41467-019-11258-x)
Supplement: Supplementary file 1 — Supplementary Information [file 41467_2019_11258_MOESM1_ESM.pdf]

Supplementary Information

Phosphorylation of MAVS/VISA by Nemo-like Kinase (NLK) for Degradation Regulates the Antiviral Innate Immune Response

Li *et al.*

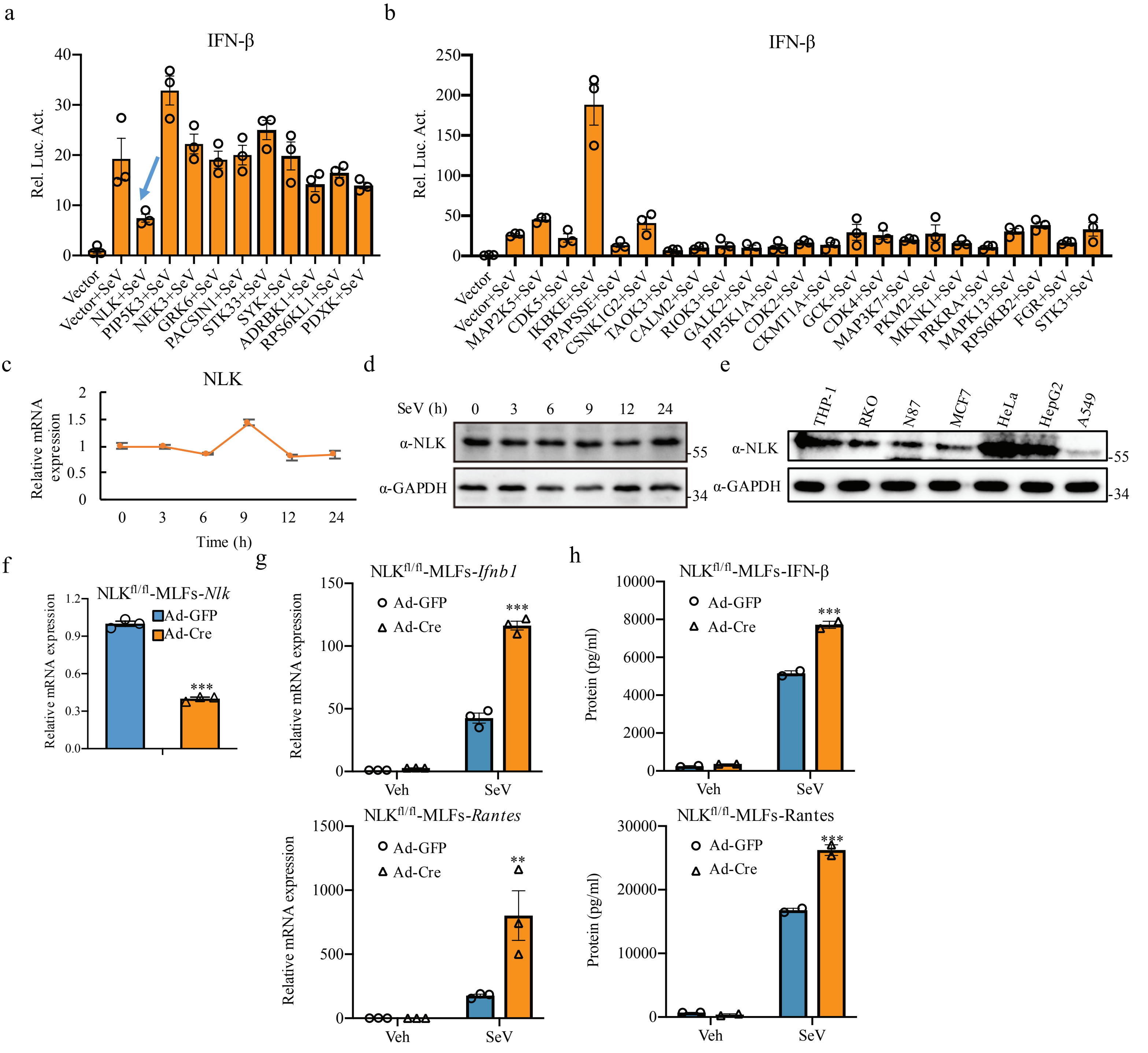

Supplementary Figure 1. NLK negatively regulates the type I interferon signaling pathway. (a, b) Screening of kinases for roles in SeV-induced IFN-β signaling using the luciferase assay. HEK293T cells were cotransfected with the IFN-β reporter (100 ng) and a kinase library expression plasmid (400 ng). After 24 hours, SeV was added to the cells for 12 hours, and reporter gene activity was then assayed using the luciferase kit. (c, d) NLK mRNA and protein levels at the indicated time points after SeV infection. HEK293T cells were infected with SeV, and mRNA and protein were harvested and analyzed using real-time PCR and immunoblotting, respectively. (e) NLK protein expression in various cell lines as determined through immunoblot analysis. Cell lysates were separated via SDS-PAGE and immunoblotted with the NLK antibody. GAPDH was used as a loading control. (f-h) NLK deficiency potentiates SeV-induced *Ifnb1* and *Rantes* gene expression in MLFs. Identification of the NLK deletion at the mRNA level (f). NLK was knocked down in NLK$^{fl/fl}$-MLFs using Ad-Cre virus, followed by SeV infection and real-time PCR analysis. (g-h). Data are representative of three (c-e) or two (f-h) independent experiments. Data are presented as the mean ± SEM (n=3 for a, b, f, g and n=2 for h). Statistical significance was analyzed by ANOVA or Student's *t*-test (***p* < 0.01, ****p* < 0.001). Source data (a-h) are provided as a Source Data file.

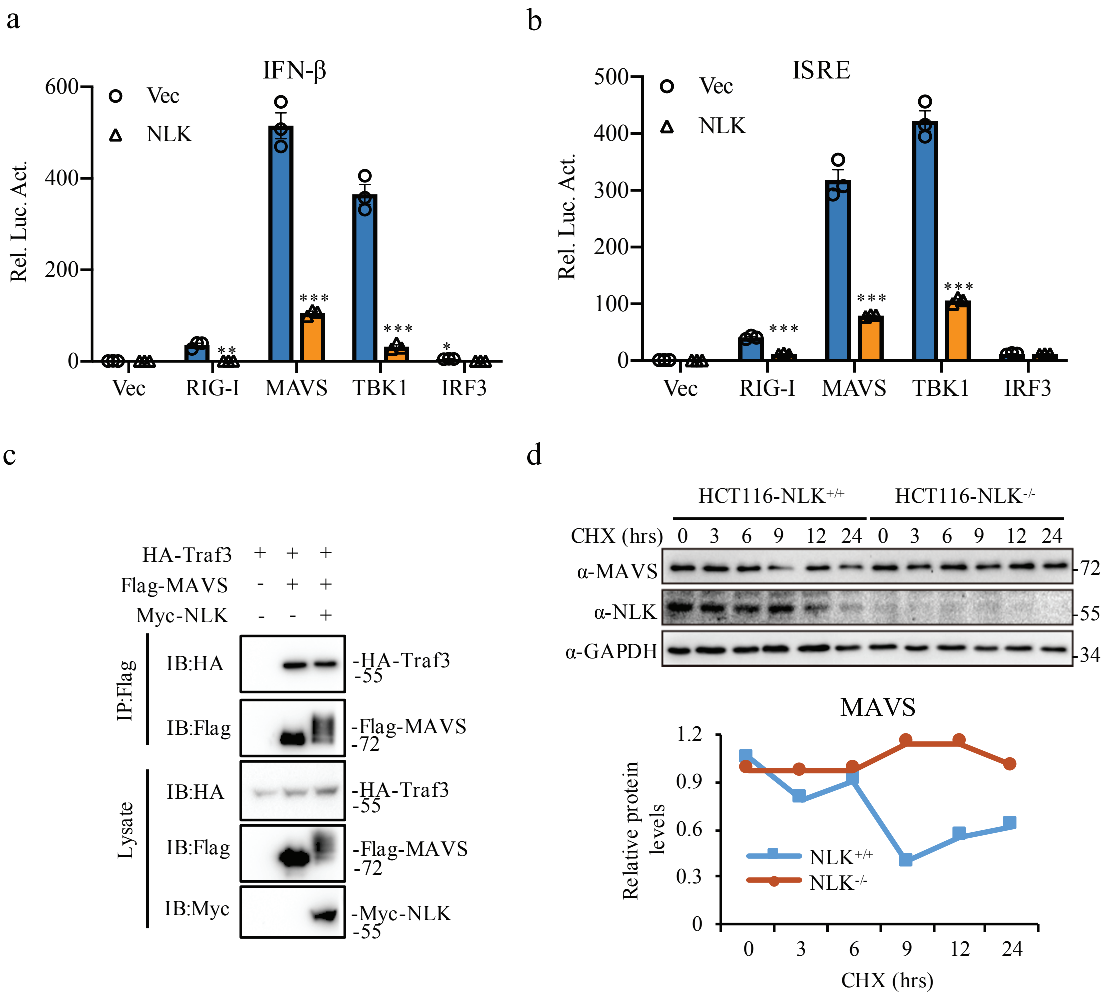

Supplementary Figure 2. NLK interacts with and degrades MAVS. (a, b) NLK inhibits SeV-induced IFN-β and ISRE activation at the TBK1 level. HEK293T cells were transfected with the indicated plasmids for 24 hours, followed by infection with SeV for 12 hours. The cells were subsequently lysed and subjected to luciferase assays. (c) Influence of NLK on the stability of the MAVS and TRAF3 complex as determined by immunoblot. HEK293T cells were transfected with the indicated plasmids for 24 hours. The cells were then lysed and subjected to immunoblot analysis using the indicated antibodies. (d) Stability of MAVS in wild-type and NLK-deficient HCT116 cells. HCT116 cells were treated with CHX for the indicated periods, and the cell lysate was subjected to immunoblotting using MAVS and NLK antibodies. The relative levels of MAVS were calculated after normalization to GAPDH and are shown in the bottom panel. Data are representative of three independent experiments. Data are presented as the mean ± SEM (n=3). Statistical significance was analyzed by ANOVA (*$p < 0.05$, **$p < 0.01$, ***$p < 0.001$). Source data (a-d) are provided as a Source Data file.

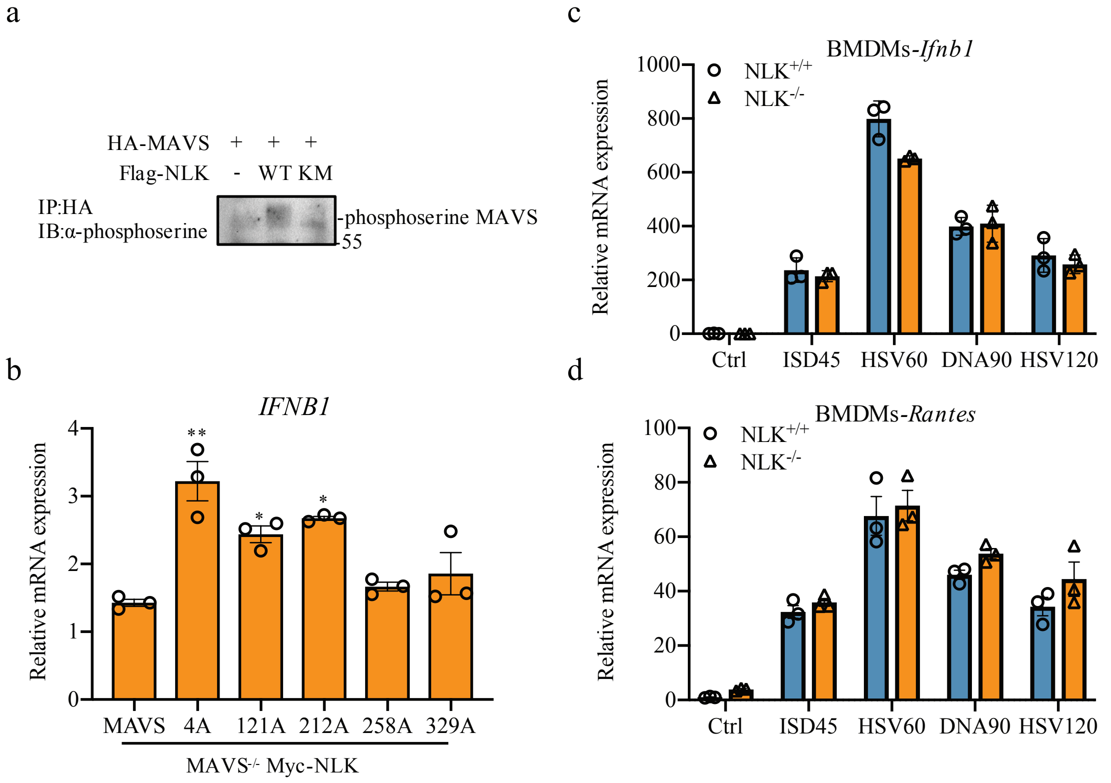

Supplementary Figure 3. NLK phosphorylates MAVS and alters proteasome-dependent degradation. (a) Phosphorylation of the MAVS protein by NLK *in vitro*. HEK293T cells were transfected with the indicated expression vectors. The protein was immunoprecipitated with the anti-HA antibody and subjected to immunoblotting using the anti-phosphoserine antibody. (b) Effects of MAVS mutants on the production of *IFNB1*. MAVS$^{-/-}$ HEK293T cells were transfected with NLK and MAVS or MAVS mutants. RNA was isolated after 36 hours and analyzed by real-time PCR. 121A: mutation of serine 121 to alanine. 212A: mutation of serine 212 to alanine. 258A: mutation of serine 258 to alanine. 329A: mutation of serine329 to alanine. 4A: mutation of serine 121/212/258/329 to alanine. (c and d) Effect of NLK deficiency on the production of antiviral cytokines in BMDMs in response to the different types of DNA ligands. BMDM cells were exposed to the indicated DNA ligands. mRNA was isolated to determine the transcription levels of *Ifnb1* and *Rantes*. Data are representative of three (a and b) independent experiments. Data are presented as the mean ± SEM (n=3). Statistical significance was analyzed by ANOVA (**p* < 0.05, ***p* < 0.01,). Source data (a-d) are provided as a Source Data file.

Supplementary Figure 4

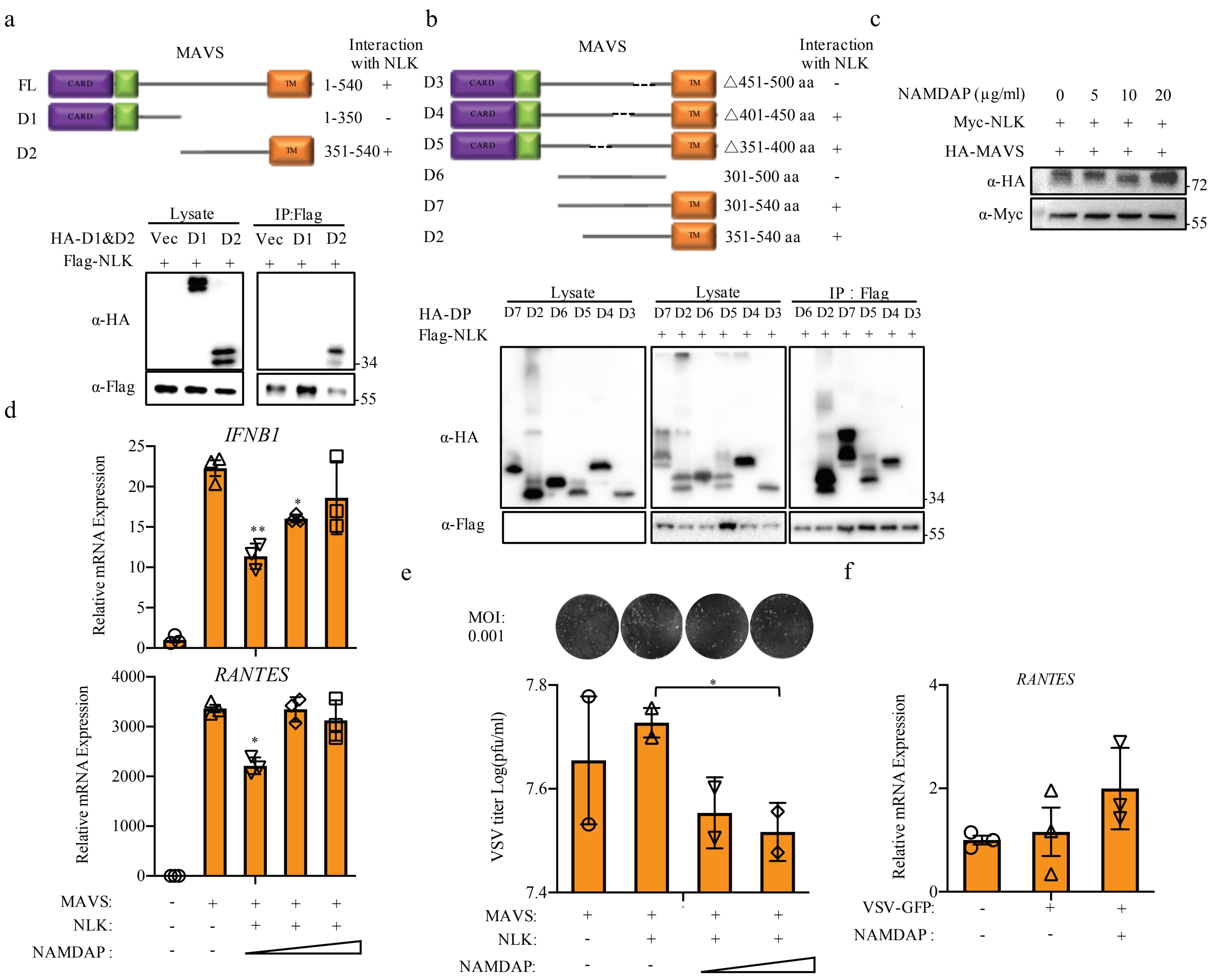

Supplementary Figure 4. A peptide from MAVS potentiates the antiviral response *in vitro* and *in vivo*. (a, b) Domain mapping of NLK and MAVS. HEK293T cells were transfected with MAVS mutants (top panel) and NLK and then subjected to immunoprecipitation using a Flag antibody followed by immunoblot analysis. (c) The effect of NAMDAP on the stabilization of MAVS in the presence of NLK. HEK293T cells expressing MAVS together with NLK were incubated with increasing amounts of NAMDAP before performing immunoblot analysis. (d) The effect of NAMDAP on *IFNB1* and *RANTES* gene transcription. HEK293T cells expressing MAVS together with NLK were incubated with increasing amounts of NAMDAP before performing real-time PCR experiments. (e) HEK293T cells expressing MAVS and NLK were incubated with VSV-GFP at an MOI of 0.001 and increasing amounts of NAMDAP. The supernatant was added to Vero cells, and the plaque assay was then performed. (f) Effect of NAMDAP on the production of antiviral cytokines *in vitro*. HEK293T cells were infected with VSV-GFP at an MOI of 0.01, and mRNA was isolated to determine the transcription level of *RANTES*. Data are representative of three independent experiments. Data are presented as the mean ± SEM (n=3 for d, f and n=2 for e). Statistical significance was analyzed by ANOVA (*$p < 0.05$, **$p < 0.01$). Source data (a-f) are provided as a Source Data file.

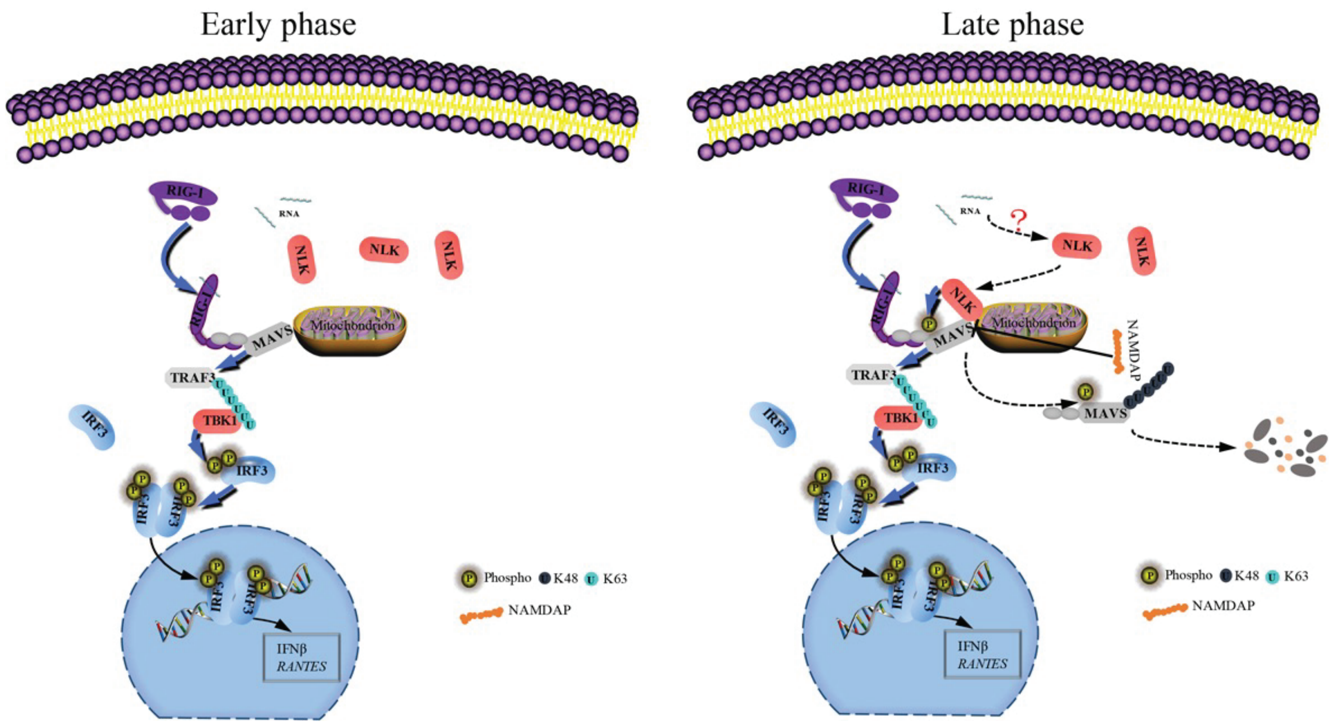

Supplementary Figure 5. Proposed model illustrating how NLK negatively regulates the type I IFN signaling pathway.

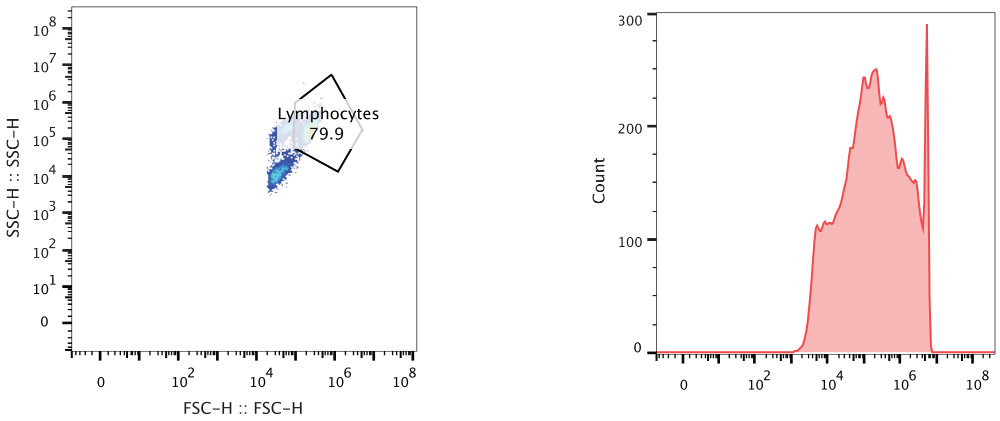

Supplementary Figure 6. The gating strategy. VSV-GFP positive cells were defined through FITC lane. The same gating strategy was applied on Figure 2c, 3f, 4d and 8e.

Supplementary Table 1. The primer sequence for RNA expression or genome typing

Genes	Primers	Primer sequences	Application
hGAPDH	Forward	5'-GAGTCAACGGATTTGGTCGT-3'	RNA expression
	Reverse	5'-GACAAGCTTCCCGTTCTCAG-3'	RNA expression
hIFN-β	Forward	5′-TGACTATGGTCCAGGCACAG-3′	RNA expression
	Reverse	5′-TTGTTGAGAACCTCCTGGCT-3′	RNA expression
hISG15	Forward	5′-AGGACAGGGTCCCCCTTGCC-3′	RNA expression
	Reverse	5′-CCTCCAGCCCGCTCACTTGC-3′	RNA expression
hISG56	Forward	5′-TCATCAGGTCAAGGATAGTC-3′	RNA expression
	Reverse	5′-CCACACTGTATTTGGTGTCTAGG-3′	RNA expression
hRANTES	Forward	5′-GGCAGCCCTCGCTGTCATCC-3′	RNA expression
	Reverse	5′-GCAGCAGGGTGTGGTGTCCG-3′	RNA expression
mIfnβ1	Forward	5′-CTGCCTTTGCCATCCAAGAG-3′	RNA expression
	Reverse	5′-TGTCTGCTGGTGGAGTTCAT-3′	RNA expression
mRantes	Forward	5′-ATATGGCTCGGACACCACTC-3′	RNA expression
	Reverse	5′-CACTTGCTGCTGGTGTAGAA-3′	RNA expression
mflox	Forward	5′-CTGTAACCACGAGGACAGCA-3′	Genome typing
	Reverse	5′-TTCTCCCTCCCTCTCTCACA-3′	Genome typing
mNlk	Forward	5′-TCTTCCGGGAATTGAAGATG-3′	RNA expression
	Reverse	5′-TCTGAGCTGAGTGGTTGAGG-3′	RNA expression
