# Supplementary material for: Phosphorylation of MAVS/VISA by Nemo-like kinase (NLK) for degradation regulates the antiviral innate immune response
Source: Nat Commun. 2019 Jul 19;10:3233. doi: 10.1038/s41467-019-11258-x (PMC6642205; doi:10.1038/s41467-019-11258-x)
Supplement: Supplementary file 3 — Reporting Summary [file 41467_2019_11258_MOESM3_ESM.pdf]

nature research

Corresponding author(s): xiaodong zhang

Last updated by author(s): Jun 22, 2019

Reporting Summary

Nature Research wishes to improve the reproducibility of the work that we publish. This form provides structure for consistency and transparency in reporting. For further information on Nature Research policies, see Authors & Referees and the Editorial Policy Checklist.

Statistics

For all statistical analyses, confirm that the following items are present in the figure legend, table legend, main text, or Methods section.

n/a	Confirmed	
☐	☒	The exact sample size (*n*) for each experimental group/condition, given as a discrete number and unit of measurement
☐	☒	A statement on whether measurements were taken from distinct samples or whether the same sample was measured repeatedly
☐	☒	The statistical test(s) used AND whether they are one- or two-sided<br>*Only common tests should be described solely by name; describe more complex techniques in the Methods section.*
☒	☐	A description of all covariates tested
☐	☒	A description of any assumptions or corrections, such as tests of normality and adjustment for multiple comparisons
☐	☒	A full description of the statistical parameters including central tendency (e.g. means) or other basic estimates (e.g. regression coefficient) AND variation (e.g. standard deviation) or associated estimates of uncertainty (e.g. confidence intervals)
☐	☒	For null hypothesis testing, the test statistic (e.g. *F*, *t*, *r*) with confidence intervals, effect sizes, degrees of freedom and *P* value noted<br>*Give P values as exact values whenever suitable.*
☒	☐	For Bayesian analysis, information on the choice of priors and Markov chain Monte Carlo settings
☒	☐	For hierarchical and complex designs, identification of the appropriate level for tests and full reporting of outcomes
☒	☐	Estimates of effect sizes (e.g. Cohen's *d*, Pearson's *r*), indicating how they were calculated

Our web collection on statistics for biologists contains articles on many of the points above.

Software and code

Policy information about availability of computer code

Data collection	prism (version 8.1.1)
Data analysis	prism (version 8.1.1)

For manuscripts utilizing custom algorithms or software that are central to the research but not yet described in published literature, software must be made available to editors/reviewers. We strongly encourage code deposition in a community repository (e.g. GitHub). See the Nature Research guidelines for submitting code & software for further information.

Data

Policy information about availability of data

All manuscripts must include a data availability statement. This statement should provide the following information, where applicable:

- Accession codes, unique identifiers, or web links for publicly available datasets
- A list of figures that have associated raw data
- A description of any restrictions on data availability

The authors declare that all data supporting the findings of this investigation are available within the article, its Supplementary Information, and from the corresponding authors upon reasonable request.

Field-specific reporting

Please select the one below that is the best fit for your research. If you are not sure, read the appropriate sections before making your selection.

☒ Life sciences ☐ Behavioural & social sciences ☐ Ecological, evolutionary & environmental sciences

Life sciences study design

All studies must disclose on these points even when the disclosure is negative.

Sample size	N/A
Data exclusions	N/A
Replication	N/A
Randomization	N/A
Blinding	N/A

Reporting for specific materials, systems and methods

We require information from authors about some types of materials, experimental systems and methods used in many studies. Here, indicate whether each material, system or method listed is relevant to your study. If you are not sure if a list item applies to your research, read the appropriate section before selecting a response.

Materials & experimental systems

n/a	Involved in the study	
☐	☒	Antibodies
☐	☒	Eukaryotic cell lines
☒	☐	Palaeontology
☐	☒	Animals and other organisms
☒	☐	Human research participants
☒	☐	Clinical data

Methods

n/a	Involved in the study	
☒	☐	ChIP-seq
☐	☒	Flow cytometry
☒	☐	MRI-based neuroimaging

Antibodies

Antibodies used	NLK (A400-046A), MAVS (A300-782A) (Bethyl, TX, USA), NLK (monoclonal antibody for IF produced by ourself), Flag (F1804), Catalase (219010) (Sigma-Aldrich, St. Louis, MO, USA), Myc (CW0299M), GAPDH (CW0101M), Tubulin (CW0098M), (CWBIO, Beijing, China), HA (51064-2-AP), COX IV(11242-1-AP), TOM20 (11802-1-AP, Proteintech, Hubei, China), p-IκBα (9246), p-IRF3 (29047) (Cell Signaling, Danvers, MA, USA) and MAVS (sc-365333 for IF), IRF3 (sc-376455) (Santa Cruz Biotechnology, Santa Cruz, CA, USA) antibodies were purchased from the indicated companies.
Validation	NLK antibody was validated by NLK deficient cell lines and mice. All the other antibodies were tested by the manufacturers.

Eukaryotic cell lines

Policy information about cell lines

Cell line source(s)	All the cell lines were come from ATCC (Zhenghe Wang and Hongbing Shu' lab previously used)
Authentication	Common used 293T and HCT116 were further identified by commpany. Murine cell lines were derived by ourself.
Mycoplasma contamination	Mycoplasma contamination was detected for common used cell lines, 293T and HCT116.<br>And there is no contamination of Mycoplasma
Commonly misidentified lines (See ICLAC register)	N/A

Animals and other organisms

Policy information about studies involving animals; ARRIVE guidelines recommended for reporting animal research

Laboratory animals	The male or female C57BL/6 mice at 6-8 weeks were used for the experiments.
Wild animals	N/A
Field-collected samples	All mice were housed under a 12:12-h light/dark cycle at controlled temperature.

Ethics oversight	All animal studies were conducted in accordance with the Guidelines of the China Animal Welfare Legislation and approved by the Committee on Ethics in the Care and Use of Laboratory Animals of Wuhan University (permit number: 15060A). All efforts were made to minimize suffering.

Note that full information on the approval of the study protocol must also be provided in the manuscript.

Flow Cytometry

Plots

Confirm that:

☒ The axis labels state the marker and fluorochrome used (e.g. CD4-FITC).

☒ The axis scales are clearly visible. Include numbers along axes only for bottom left plot of group (a 'group' is an analysis of identical markers).

☒ All plots are contour plots with outliers or pseudocolor plots.

☒ A numerical value for number of cells or percentage (with statistics) is provided.

Methodology

Sample preparation	After 16 hours of GFP-VSV infection, the indicated cells were harvested and resuspended in 0.5 mL phosphate-buffered saline (PBS)
Instrument	The samples were analyzed by flow cytometry (Cytoflex, Beckman Coulter, Fullerton, CA, USA)
Software	The flow cytometry data were collected by cytoexpert (Beckman Coulter, Fullerton, CA, USA) and analyzed using FlowJo software (TreeStar, Ashland, OR, USA)
Cell population abundance	around 10000-20000 cells were collected
Gating strategy	According to the intensity of GFP

☒ Tick this box to confirm that a figure exemplifying the gating strategy is provided in the Supplementary Information.
